# Observations on Diffused Aneurism

**Published:** 1827-06

**Authors:** 

**Affiliations:** Surgeon of the Macclesfield Dispensary.


### ANEURISM.

*Observations on Diffused Aneurism.* By —— Dickinson, Esq. Surgeon of the Macclesfield Dispensary.

There are few cases in surgery which require more accurate discrimination, or more decisive and prompt treatment, than Aneurisms; and there are few cases, happily, over which modern science has cast more light, or in the management of which the triumph of our art has been more conspicuous. But, vast as has been the improvement of this branch of surgery from the time when John Hunter, guided by scientific principles, first devised his new mode of operation, to the period when Mr. Wardrop revived and perfected the method of *ultra* ligature, still there is much scope for investigation upon this interesting subject.

The points to which I wish to call attention are, first, the very rapid and destructive effects produced by this disease upon the bony structure of our frame; and, secondly, the question as to the proper mode of treatment in those cases where, from neglect or other circumstances, the disease has passed through its simple form, and, by bursting and discharging its contents into the intermuscular spaces or cellular structure, has assumed that state which has been denominated Diffused Aneurism.

That the constant pulsation of an aneurism is capable of causing the absorption of bone, must be familiar to every one; but the shortness of time in which that effect may occasionally be produced, I am inclined to believe is not so generally known. I here beg to remark, that there is a peculiarity in our system with respect to the influence of arterial pulsation upon bone, which is apt to escape notice from its constant occurrence: it is not, however, of much practical importance, but may serve to prove the admirable wisdom of those laws which govern our corporeal economy. Though the pulsation of an aneurism will excite absorption of bone, yet the same result is never discovered from the continual beating of an artery when in close *natural* proximity with bone: for instance, the arteria meningea media frequently runs through a bony channel in its course, and, from determination of blood to the head, must often act with much force against its osseous boundary, yet we never find absorption of bone the consequence. The like observation will apply wherever an artery passes through a bony canal. It would seem that our constitution is guarded against the evil influence of natural operations, though similar actions will cause disease when applied in situations, and under circumstances, contrary to the general order of our frames.

Absorption of bone has been considered an ordinary, but slow, effect of aneurismal pressure; but, in a case which I shall relate, the complete destruction of four inches of the fibula was accomplished in a very few weeks. This circumstance demands notice, as it may prevent surgeons from allowing delay of operation whenever the disease is known to exist. There is also another motive for promptitude, deducible from the same case, namely, the short interval which sometimes elapses between the first formation of the disease and the period when it assumes the character of diffused aneurism. This last reason for vigorous measures may not, perhaps, at first sight appear very imperative; but I trust I shall be able to establish the fact that the most important results to the patient depend upon the disease being met in its early and simple form.

In the instance I shall mention, the term of a few weeks witnessed the commencement of simple aneurism, and its termination in diffused aneurism. That such rapid progress is not generally understood to be a character of the disease, I may be allowed to presume, from the circumstance of Sir Anthony Carlisle having, in a case reported in the 11th volume of the Lancet, expressed his disbelief of a tumor being diffused aneurism, from the account of its early rupture; and yet that rupture was not at so early a period of the complaint as in the case which I have to record. To the case in the Lancet referred to above, I shall have occasion to return presently. My immediate object is to discuss the question of the safest and best plan when simple aneurism has become diffused.

When, then, we find the walls of the sac have given way, and blood is extensively diffused into the limb, what are we to do? That the vital powers of a limb are much oppressed by an extensive engorgement of its intermuscular and cellular spaces, every one must have observed; that gangrene or sloughing not unfrequently occur, must be allowed. Shall we then render such termination much more probable by tying the main trunk of the artery, thus almost annihilating the vitality of the part? I think the general answer will be in the negative. Moreover, there are formidable dangers, even should we be fortunate enough to preserve the life of the limb; for, after the ligature of the artery, a partial circulation is established, by which a return of a pulsatory action is effected in the sac: this, in simple aneurism, is of little consequence, as the supply of blood is not sufficient to prevent coagulation of its contents and obliteration of the sac. In diffused aneurism, however, the case is widely different: there the blood is poured out into a space without definite boundary, unless the external integuments be considered as such; and though it may be objected, that when the limb will hold no more the effusion of blood will cease, yet have we not great reason to fear that, when the pressure is sufficient to block up the mouth of the bleeding vessel, or to compress its trunk, the circulation will also be suppressed in the other vessels which have to carry on the life of the part? and if so, how much greater is the risk of gangrene. But suppose the case to be altered from this less fearful state of diffused aneurism, to that in which the sac has burst, or has been opened externally, here the danger is still greater; for we have before us the hazard of a fatal hemorrhage, unless by external pressure, or the tourniquet, the flow of blood be commanded. It need scarcely be remarked how little chance of life a limb would retain under such circumstances. In several instances, the patient has fallen a sacrifice to the well-intended attempt to preserve the limb; and in numerous cases amputation has become requisite, from the unrestrainable bleeding, at the time of operation, or in a few days after. This misfortune occurred in the case of Sir Anthony Carlisle, already referred to.

From all these circumstances, it appears to me to be a question well worthy the consideration of the profession, whether amputation be not, in almost every instance of diffused aneurism, the safest and most proper remedy. For the view here taken, I must confess myself much indebted to an anonymous correspondent in the Lancet, volume 5th, No. 9, whose arguments had much influence in deciding the mode of treatment I have now to narrate.

On Monday evening, March 27th, 1827, I was requested by a surgeon to see a case of tumor, which he supposed to be fungus hæmatodes. He stated that it was situated upon the upper and outer part of the left leg; that he had opened it, and that some hemorrhage had ensued, together with the discharge of a quantity of brain-like substance. Upon the following morning I saw the patient, a little girl about fourteen years of age, and found the limb generally distended from the knee to the ankle, with a considerable tumor upon the part mentioned. Upon introducing my finger into the opening which had been made into this tumor, I found it to be formed by a large bag of coagulated blood, through which I could feel the posterior part of the tibia very slightly covered. On withdrawing my finger, there was a profuse gush of coagula, followed by a stream of arterial blood. There could be no doubt that the disease was diffused aneurism of the peroneal or posterior tibial artery.

The account given was, that the girl had complained of aching pain of the leg for twelve or thirteen weeks past, which was considered at first what is popularly called *thrift*. When the swelling was perceived, it was mistaken for incipient abscess, and fomentations and poultices were applied for the purpose of promoting suppuration. She had been prevented from walking for about six weeks. An incision was made into it on the 8th of March, when there was a discharge of about two table-spoonfuls of blood. On the 14th, a fresh incision was effected, when a large bleeding followed; and upon the 21st the tumor burst externally, and a profuse hemorrhage was the consequence, which continued in a lesser degree two nights and a day, and was at last checked by a tent of lint. On the 27th, the wound was enlarged, and a violent bleeding ensued, which was with great difficulty restrained. The surgeon assured me he had never felt any pulsation; but at a subsequent period the little patient stated to me that the swelling had "at times panted like her heart."

As the poor child was much reduced by these repeated hemorrhages, and as a more violent bleeding might at any moment occur, no time was to be lost, and I immediately declared that ligature of the artery, or amputation of the limb, ought to be performed that very day; but, as such a measure might appear abrupt in a case which had not hitherto alarmed the friends of the patient, I requested a consultation with my friend Mr. Cockson, who in a short time joined us. In our consultation it was considered that, owing to the very extensive extravasation of blood into the intermuscular spaces, and the feeble state of the girl,—taking into account, also, the unfavourable results of several operations of ligature of the artery in similar cases in London, Portsmouth, and elsewhere,—it would be safer to amputate than to attempt to save the limb. To this determination we were led by the conviction that such extensive engorgement would require much vital power to support the consequent suppuration, even in a limb whose circulation was uninterrupted, but that, when the main trunk was tied, in all probability gangrene would ensue; besides, as there is usually a partial return of circulation after the operation of ligature, here we had great reason to fear that such returning circulation would cause an unmanageable hemorrhage, by which the patient would either be drained to death, or amputation would be rendered unavoidable at a period when the constitution would be in a weak and irritable condition. From these considerations amputation was decided upon, and immediately performed. The vessels of the stump were found healthy.

Upon examination of the limb, the saphænus nerve was seen tightly stretched over the gastrocnemius, upon the separation of which muscle from the condyles of the femur, a large bag was discovered filled with coagula, mingled with a brainy matter, to the amount of nearly a quart. Nothing like the regular tissue of a sac was found, but the surface of the muscles assumed more the appearance of the cavity of an old ulcer or abscess. At that part of the tibia to which the head of the fibula is attached, only a slight shell of bone was left; and, upon a further examination, it was perceived that the entire cylinder of the fibula had been destroyed to the extent of about four inches. The brainy substance we supposed to have been formed by the admixture of the vestiges of the bone with the coagulable lymph of the blood. One circumstance may be noted as confirmatory of the accounts given of this disease: though the bone had been so extensively absorbed, not a single drop of matter had been secreted in the process.

Upon tracing the vessels, the disease was found to have been situated in the course of the posterior tibial artery, the superior ruptured portion of which was seen loose in the sac. The lower portion was not discovered.

At the time of writing this account, the little girl is doing well, and the stump nearly closed.

*Macclesfield; April,* 1827.

